# Predicting Current Glycated Hemoglobin Values in Adults: Development of an Algorithm From the Electronic Health Record

**DOI:** 10.2196/10780

**Published:** 2018-10-22

**Authors:** Brian J Wells, Kristin M Lenoir, Jose-Franck Diaz-Garelli, Wendell Futrell, Elizabeth Lockerman, Kevin M Pantalone, Michael W Kattan

**Affiliations:** 1 Division of Public Health Sciences Department of Biostatistics and Data Science Wake Forest University School of Medicine Winston-Salem, NC United States; 2 Department of Physiology and Pharmacology Wake Forest University School of Medicine Winston-Salem, NC United States; 3 Clinical and Translational Science Institute Wake Forest University School of Medicine Winston-Salem, NC United States; 4 Department of Internal Medicine Loyola University Medical Center Maywood, IL United States; 5 Endocrinology and Metabolism Institute Department of Endocrinology, Diabetes and Metabolism Cleveland Clinic Cleveland, OH United States; 6 Lerner Research Institute Department of Quantitative Health Sciences Cleveland Clinic Cleveland, OH United States

**Keywords:** electronic health records, risk prediction, clinical decision support, hemoglobin A1c, diabetes

## Abstract

**Background:**

Electronic, personalized clinical decision support tools to optimize glycated hemoglobin (HbA_1c_) screening are lacking. Current screening guidelines are based on simple, categorical rules developed for populations of patients. Although personalized diabetes risk calculators have been created, none are designed to predict current glycemic status using structured data commonly available in electronic health records (EHRs).

**Objective:**

The goal of this project was to create a mathematical equation for predicting the probability of current elevations in HbA_1c_ (≥5.7%) among patients with no history of hyperglycemia using readily available variables that will allow integration with EHR systems.

**Methods:**

The reduced model was compared head-to-head with calculators created by Baan and Griffin. Ten-fold cross-validation was used to calculate the bias-adjusted prediction accuracy of the new model. Statistical analyses were performed in R version 3.2.5 (The R Foundation for Statistical Computing) using the rms (Regression Modeling Strategies) package.

**Results:**

The final model to predict an elevated HbA_1c_ based on 22,635 patient records contained the following variables in order from most to least importance according to their impact on the discriminating accuracy of the model: age, body mass index, random glucose, race, serum non–high-density lipoprotein, serum total cholesterol, estimated glomerular filtration rate, and smoking status. The new model achieved a concordance statistic of 0.77 which was statistically significantly better than prior models. The model appeared to be well calibrated according to a plot of the predicted probabilities versus the prevalence of the outcome at different probabilities.

**Conclusions:**

The calculator created for predicting the probability of having an elevated HbA_1c_ significantly outperformed the existing calculators. The personalized prediction model presented in this paper could improve the efficiency of HbA_1c_ screening initiatives.

## Introduction

Many prediction tools have been created to assess the risk of undiagnosed diabetes and related outcomes such as impaired glucose tolerance, prediabetes, risk of future diabetes, and hyperinsulinemia. Most of these tools are not practical in the setting of the electronic health record (EHR) because they include predictor variables not readily available in structured formats [1-20]. Examples of impractical variables include waist circumference, fasting time, physical activity, review of systems, diet, pregnancy-related variables, and detailed ethnicity. Tools typically leverage fasting glucose level as a predictor, which is simple to obtain in practice, but documentation of fasting time in a structured fashion in EHRs is generally absent. The authors identified two tools that accurately predict the presence of diabetes using structured variables routinely present in the EHR [21,22].

Current guidelines from the United States Preventive Services Task Force (USPSTF) recommend screening for abnormal blood glucose in adults aged 40 to 70 years who are overweight or obese. The USPSTF acknowledges that patients with other high-risk characteristics (eg, family history of diabetes, personal history of gestational diabetes) may need to be screened sooner but this is left up to the physician’s discretion [23]. The guidelines published by the American Diabetes Association (ADA) recommend glucose screening of adult patients with an elevated body mass index (BMI; ≥25 kg/m^2^) plus another risk factor (eg, hypertension, physical inactivity, family history of diabetes) at any age and for all patients beginning at 45 years of age at 3-year intervals [24].

Current approaches do not take advantage of advanced statistical modeling. Hyperglycemia risk prediction with simultaneous consideration of numerous independent variables and nonlinear effects is statistically ideal in the context of a multifactorial pathology. Creating strict cutoffs of individual variables or combinations of a limited number of variables for clinical guidelines does not take advantage of what can now be reasonably achieved. The USPSTF and ADA guidelines encourage physician judgment in the application of glucose screening but do not provide specific guidance. Simplified classification methods used in cancer have been notoriously poor at discriminating between high- and low-risk patients [25]. Moreover, many existing models for predicting hyperglycemia risk are also likely reducing their prediction accuracy by categorizing continuous variables, which reduces granularity and may miss potentially complex associations between a continuous variable and the outcome. This issue was highlighted by Kattan [26] when he showed that traditional regression techniques that incorporated restricted cubic splines to reduce linearity assumptions were found to produce more accurate risk prediction models when compared with classification methods such as classification and regression trees and artificial neural networks.

Predicting the date of onset of hyperglycemia is difficult due to the lack of symptoms early in the course of the disease and inconsistent testing and/or documentation in clinical practice (particularly in a structured fashion). Previous studies indicate that the onset of type 2 diabetes frequently occurs more than 5 years before diagnosis [27,28]. In contrast, blood measurements of glycated hemoglobin (HbA_1c_) provide an easy and accurate method for determining current mean glycemia over the previous 8 to 12 weeks without the need for fasting. HbA_1c_ testing is standardized according to specifications defined by the National Glycohemoglobin Standardization Program (NGSP). HbA_1c_ levels are the primary blood marker used for guiding the management of type 2 diabetes, and the ADA has approved HbA_1c_ testing for diabetes screening [24]. The increasing use of HbA_1c_ as a screening tool in patients without prediabetes or diabetes provides data for prediction modeling from EHR records.

The authors strongly believe that the identification of patients with elevated HbA_1c_ is important clinically despite previous studies that have not shown a mortality benefit from screening for diabetes [29]. The early detection of elevated blood sugar can have other significant benefits:

- Behavioral counseling can lead to reductions in cardiovascular disease risk [30].
- Treatment of prediabetes, which affects approximately 35% of the adult population in the United States [31], has been shown to delay progression to diabetes [32].
- Diabetic-specific retinopathy is present in up to 21% of patients with newly diagnosed type 2 diabetes [33], while peripheral neuropathy and nephropathy are present in 21.5% and 26.5%, respectively, of patients with undiagnosed diabetes [34]. Aggressive blood sugar and blood pressure control among patients with diabetes reduces the risk of microvascular complications [35,36].
- Early detection of diabetes allows for the allocation of proven preventive strategies (eg, fundoscopic screening for retinopathy, pneumococcal vaccination, screening for nephropathy, and aggressive prevention of cardiovascular disease) [37].
- Appropriate documentation of elevated blood sugar and diabetes allows health systems and payers to improve the risk stratification of patients and increases the potential pool of patients available for participation in clinical research.

Therefore, an accurate tool for predicting the current probability that a specific patient has an elevation in HbA_1c_ levels would constitute a major advancement in finding patients with the most probable need of screening interventions. To address this gap, we created a calculator for predicting the probability that a given patient with no history of diabetes or elevated blood sugar currently has an elevated HbA_1c_ value (≥5.7%). This cutoff was chosen because it corresponds with the current guidelines published by the ADA that indicate values <5.7% are considered to be normal. Importantly, the calculator presented in this paper was restricted only to structured variables typically available in EHRs. This focus on common structured variables will enable the tool to be integrated into EHRs for implementation.

## Methods

This study was conducted on all adult patients who have undergone HbA_1c_ testing prior to evidence of hyperglycemia (random blood sugar ≥200 mg/dL), any diabetes-related diagnostic code, or prescription for an antihyperglycemic medication. Data were extracted from the Epicare EHR at Wake Forest Baptist Medical Center in Winston-Salem, North Carolina, for the dates between September 2012 and September 2016. The study was approved by the institutional review board and granted a waiver of informed consent. Data were limited to structured data located in these areas of the EHR: encounter diagnoses, problem list, past medical history, procedures, prescriptions, vital signs, demographics, social history, and laboratory values. Candidate predictor variables were chosen based on their theoretical association with hyperglycemia. Textbox 1 shows a list of the candidate predictor variables included in the complete statistical model.

Independent variables were defined on the date of the HbA_1c_ of interest. For missing continuous variables (eg, systolic blood pressure), the most recent prior value was used instead. Patients completely lacking values for independent variables were excluded. The investigators did not impute missing data because it was felt that imputation would not be appropriate at the point of implementation. Comorbidities were considered to be present if the patient had any structured instances of the diagnostic code on or before the date of the first HbA_1c_. Medications with start dates on or before the date of the HbA_1c_ and end dates on or after the date of HbA_1c_ were considered to be active. Medication order dates were used when the start dates were missing. Medications missing both start and order dates were excluded. Medication categories (eg, antihyperglycemics) were provided by First Databank Inc. Multiple logistic regression was used to model the association between the independent variables and the outcome of HbA_1c_ ≥5.7%. Continuous variables were fit using restricted cubic splines with 3-knots. Due to collinearity, the model could not be fit with the simultaneous inclusion of serum non–high-density lipoprotein and high-density lipoprotein. Therefore, high-density lipoprotein was removed from the complete model. The model was reduced using Harrell’s model approximation method [39]. For parsimony, the diagnosis of obesity variable was removed after variable selection. The diagnosis had little impact on the prediction accuracy and was redundant since BMI is also in the model. The reduced model was compared head-to-head with the calculators created by Baan and Griffin [21,22]. The head-to-head comparisons were performed using 10-fold cross-validation in order to calculate the bias-adjusted prediction accuracy of the new model. Prediction model metrics included measures of discrimination (concordance statistic), calibration (calibration curves), and decision curves [40]. Statistical analyses were performed in R version 3.2.5 (R Foundation for Statistical Computing) using the rms (Regression Modeling Strategies) package.

**Candidate variables in the complete model prior to variable selection.**

Laboratory measurements:

- Serum triglycerides
- Random blood glucose
- Serum non–high-density lipoprotein
- Serum high-density lipoprotein (dropped due to an inability to fit model)
- Serum total cholesterol
- Estimated glomerular filtration rate (estimated from serum creatinine using the modified Chronic Kidney Disease Epidemiology Collaboration formula [38])

Active prescription medication categories:

- Antihypertensive
- First generation antipsychotic
- Second generation antipsychotic
- 3-hydroxy-3-methyl-glutaryl-*coenzyme A* reductase inhibitor (statin)
- Fibrate
- Valproic acid
- Beta-blocker
- Thiazide diuretic
- Niacin
- Oral glucocorticoid
- Protease inhibitor
- Nucleoside reverse transcriptase inhibitor
- Oral contraceptive
- Injectable medroxyprogesterone acetate
- Cyclosporine
- Sirolimus
- Tacrolimus

Diagnosis codes (see Multimedia Appendix 1):

- Hypertension
- Ischemic heart disease
- Peripheral vascular disease
- Neuropathy
- Obesity
- Hyperlipidemia

Vital signs:

- Systolic blood pressure
- Diastolic blood pressure
- Body mass index

Demographics:

- Race
- Age
- Gender

Family history:

- Number of first degree relatives with diabetes

Social history:

- Smoking status

## Results

**Figure 1:**
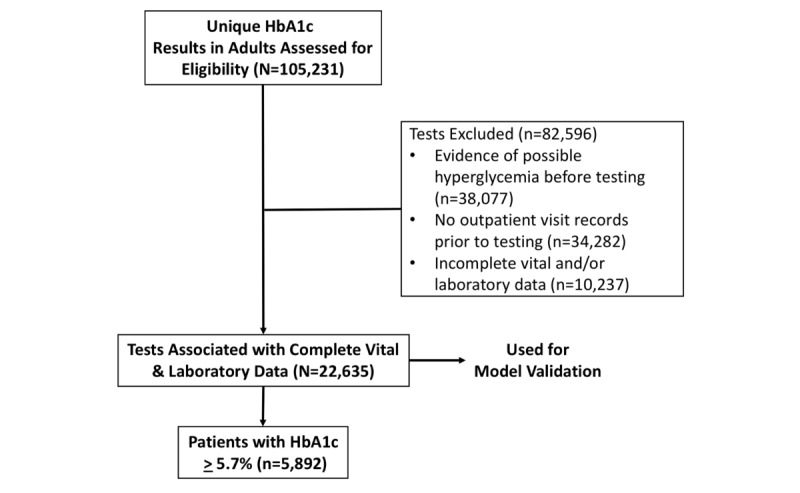
Data flowsheet. HbA_1c_: glycated hemoglobin.

The record search identified 22,635 patients for model building and validation of which 26% were found to have an elevated HbA_1c_ (≥5.7%). Figure 1 shows the number of patients included and excluded from the model building.

The final model included the following 8 variables ordered from most important to least important: age, BMI, random glucose, race, serum non–high-density lipoprotein, serum total cholesterol, estimated glomerular filtration rate (eGFR), and smoking status. Table 1 shows descriptive statistics for the variables included in the final model by HbA_1c_ results. As expected, patients found to have elevated HbA_1c_ levels were older, had higher BMI, lower eGFR, and higher random glucose values.

The coefficients along with instructions for calculating the probability of an elevated HbA_1c_ (≥5.7%) and sample calculations for 2 patient scenarios are shown in Multimedia Appendix 2.

The 3 models were compared in their ability to accurately rank patients according to risk as measured by the concordance statistic (c-stat) and bias-adjusted using 10-fold cross-validation. The current model (c-stat 0.765, 95% CI 0.762 to 0.769) demonstrated statistically significant improvements in discrimination when compared to the models created by Baan (c-stat 0.637, 95% CI 0.633 to 0.641) and Griffin (c-stat 0.668, 95% CI 0.665 to 0.672).

**Figure 2:**
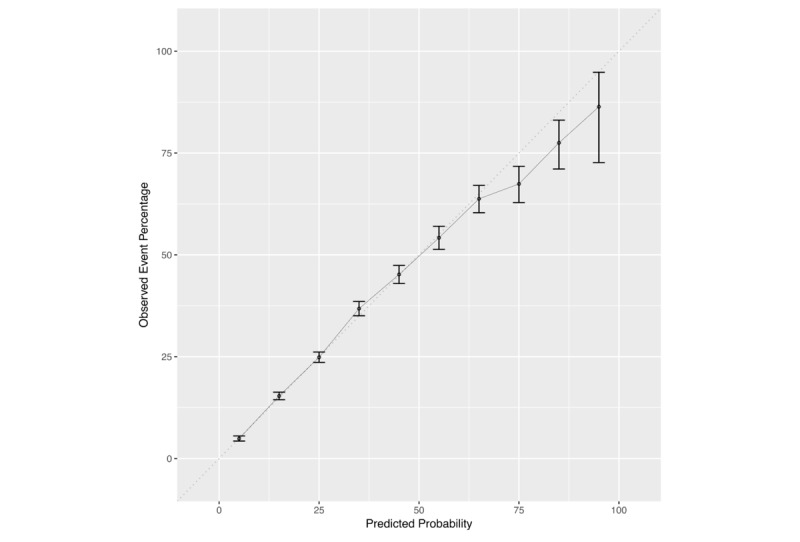
Calibration curve of the new model for predicting glycated hemoglobin ≥5.7%.

The calibration curve shown in Figure 2 reveals that the current model is well calibrated. The predicted probabilities tend to overestimate risk at the right tail of the distribution, but the wide confidence intervals allude to the scarcity of the data at these extreme high levels of risk. Error bars represent the 95% confidence interval around the point estimate.

**Figure 3:**
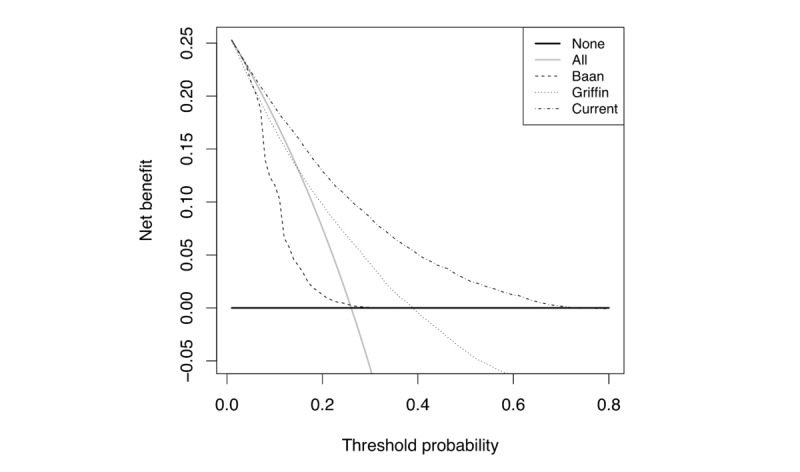
Decision curve analysis.

Decision curves are displayed in Figure 3 and also demonstrate the superiority of this model. Our model shows a net benefit up to a probability of 0.73 for an elevated HbA_1c_ (≥5.7%) without significant net harms above this threshold. This model confers a net benefit that is equal to or greater than the net benefit offered by the other models at all probability thresholds.

**Table 1:** Descriptive statistics by glycated hemoglobin outcome.

Characteristics		HbA_1c_^a^ <5.7% (n=16,743)	HbA_1c_ ≥5.7% (n=5892)	*P* value
Age (years) mean (SD)		48.1 (15.4)	54.8 (14.0)	<.001
**Race, n (%)**				<.001
	Black	3692 (22.05)	2183 (37.05)	
	Other	1178 (7.00)	487 (8.30)	
	White	11873 (70.91)	3222 (54.68)	
BMI^b^ (kg/m^2^), mean (SD)		30.1 (7.44)	33.0 (8.41)	<.001
**Smoking status, n (%)**				<.001
	Current smoker	2747 (16.41)	1393 (23.64)	
	Former smoker	3867 (23.10)	1480 (25.11)	
	Never smoker	10129 (60.50)	3019 (51.23)	
eGFR^c^ (mL/min/1.73 m^2^), mean (SD)		92.0 (33.0)	87.9 (30.8)	<.001
Random blood glucose (mg/dL), mean (SD)		88.4 (12.7)	96.1 (16.0)	<.001
Non-HDL^d^ cholesterol (mg/dL), mean (SD)		135 (37.4)	144 (41.7)	<.001
Total cholesterol (mg/dL), mean (SD)		186 (39.4)	192 (43.1)	<.001

^a^HbA_1c_: glycated hemoglobin.

^b^BMI: body mass index.

^c^eGFR: estimated glomerular filtration rate, calculated using the Chronic Kidney Disease Epidemiology Collaboration formula (CKD-EPI) [38].

^d^HDL: high-density lipoprotein.

## Discussion

### Principal Findings

The calculator created for predicting the probability of having an elevated HbA_1c_ significantly outperformed the existing calculators. It should be noted that the calculators created by Baan and Griffin were designed for predicting current glucose tolerance test results and were not specifically calibrated to predict HbA_1c_ values. However, any potential issues with calibration should not impact the ability to discriminate patients according to risk. The authors chose not to develop a simple risk score, which would be easier to calculate without a computer but would be less accurate and would not provide an absolute probability. One of the benefits of using multivariable regression over many machine learning methods is that the mathematical output of this model can be integrated into an EHR using common mathematical operations. In contrast, classification-based methods like random forest, artificial neural networks, and classification and regression trees would increase the complexity of implementation by requiring separate software outside the EHR to calculate probabilities. The movement of data into and out of the EHR also raises concerns about security and privacy.

### Limitations and Strengths

Limitations of the study include the lack of external validation. The model was validated internally using resampling and may not reflect the prediction accuracy that would be achieved in a prospective fashion at the current institution or when validated in a different health system. However, the authors used 10-fold cross-validation in which patients in the test data for each fold were not used to build the model. Another limitation pertains to the lack of data from outside health systems. Patients may have additional medication or laboratory results outside of the health system that could alter the predicted risk or change the patient’s status in terms of hyperglycemia. In order to ensure that patients have a minimal amount of data to guide the calculator’s creation, the investigators required that patients had at least one value for each of the independent variables. Future research and quality improvement projects may need to query patients about health history prior to implementation.

A relatively small proportion of historical HbA_1c_ tests were appropriate for use in model building. Some of the tests were obtained before the installation of a comprehensive EHR and, therefore, accompanying information like vital signs were not available. Many of the tests were obtained in patients who already had evidence of possible hyperglycemia, some of whom were already being treated with antidiabetic medication. These patients would be inappropriate to use for the creation of a model aimed at patients with unknown glucose status. Limiting the model building and validation dataset to patients with complete data further reduced the sample size from 32,872 to 22,635. The authors chose not to impute the missing values given the adequate number of patients with complete data. In addition, the authors are not convinced that imputation would be acceptable to patients and providers when the model is implemented into practice. Patients lacking common variables used in the model such as BMI and blood pressure values are probably very new to the health system or are seeking their usual care elsewhere. Serum creatinine and lipid measurements are routinely obtained in clinical practice, especially among older adults. Patients without any creatinine or lipid measurements are likely to be younger and less likely to be at risk for diabetes. The authors felt it was important to identify a population for model building that matches the future population where the model will be implemented. Despite the restrictions on data inclusion, the dataset contained >5000 patients with the outcome of interest. The size of this dataset is large compared to most similar studies conducted prior to the adoption of EHRs and is more than adequate for regression modeling. Harrel [39] has proposed that 7 to 10 outcomes for each degree of freedom are necessary to prevent overfitting when building a regression equation. The model created in this study contains 28 degrees of freedom, which could have safely been built from a dataset containing only 196 to 280 outcomes according to the aforementioned heuristic. A feasibility analysis was conducted among patients in the Department of Family and Community Medicine, and it was determined that approximately 20% of the adult patients seen in the past 3 years would be appropriate for application of the tool.

Imprecision in the measurement of HbA_1c_ levels could have negatively impacted the model building and could decrease the prediction accuracy of the model upon implementation. Wake Forest Baptist Medical Center is not a certified member of the NGSP but maintains accreditation by the Clinical Laboratory Improvement Amendments Program (identification number 34D0664386). The Wake Forest Baptist Medical Center’s core laboratory performs HbA_1c_ testing using ion exchange high performance liquid chromatography, which is highly precise and constituted the vast majority of HbA_1c_ measurements used to create the data for this study. However, the investigators did not exclude HbA_1c_ measurements obtained using different methods at other locations in the health system (eg, point-of-care testing), which likely introduced variability in the HbA_1c_ measurements. Comorbid conditions such as iron deficiency anemia can lead to HbA_1c_ measurements that do not accurately reflect average blood glucose levels [41]. Despite the potential negative impact of imprecise or inaccurate HbA_1c_ measurements, the prediction model performed very well.

### Conclusions

Improving the efficiency of diabetes screening should be of great interest in the United States given the increased use of value-based care contracts. Health systems could use our model for diabetes screening initiatives in a variety of ways. The decision curves suggest that using the new algorithm to guide HbA_1c_ testing would provide a net benefit between probabilities of 0.01 to 0.71. The authors will conduct a targeted screening study in which patients with a predicted risk of an elevated HbA_1c_ ≥50% will be notified directly regarding their elevated risk. Coupled with standing laboratory orders, this direct-to-patient design would enable patients to undergo HbA_1c_ testing prior to a physician visit. This is particularly important for patients with infrequent in-person visits. The hope is that patients with subsequent elevations in HbA_1c_ would be more likely to re-engage with the health system.

In summary, the risk equation created in this study is optimized for integration within an EHR and outperforms other similar models. Future research will attempt to integrate the risk calculator into clinical workflows, examine the ability of the calculator to predict risk in other health systems, and evaluate the potential economic savings of using this model for diabetes screening.

## Appendix

Multimedia Appendix 1 Diagnostic codes.

Multimedia Appendix 2 Instructions for calculating the probability of an elevated glycated hemoglobin and sample calculations.

## Abbreviations

ADA: American Diabetes Association
CKD-EPI: Chronic Kidney Disease Epidemiology Collaboration formula
c-stat: concordance statistic
eGFR: estimated glomerular filtration rate
EHR: electronic health record
HbA _1c_: glycated hemoglobin
NGSP: National Glycohemoglobin Standardization Program
USPSTF: United States Preventive Services Task Force
