# Supplementary material for: Predicting Current Glycated Hemoglobin Values in Adults: Development of an Algorithm From the Electronic Health Record
Source: JMIR Med Inform. 2018 Oct 22;6(4):e10780. doi: 10.2196/10780 (PMC6231807; doi:10.2196/10780)
Supplement: Multimedia Appendix 1 [file medinform_v6i4e10780_app1.pdf]

*Table 2***.** Diagnostic codes

ICD 9

hypertension	ischemic heart disease	peripheral vascular disease	neuropathy	obesity	hyperlipidemia	diabetes or hyperglycemia
401	410	440.2	249.60	278.0	272.0	250
402	411	440.3	249.61		272.1	249
403	412	443.9	250.60		272.2	648.8
404	413		250.61		272.3	790.2
405	414		250.62		272.4	V12.21
	996.03		250.63			
	V45.81		356.9			
	V45.82		357.2			
	429.2					

ICD 10

hypertension	ischemic heart disease	peripheral vascular disease	neuropathy	obesity	hyperlipidemia	diabetes or hyperglycemia
I10	I20.0	I70.2	E08.4	E66	E78.0	E10
I11	I20.8	I70.3	E09.4		E78.1	E11
I12	I20.9	I70.4	E10.4		E78.2	E13
I13	I21	I70.5	E11.4		E78.3	R73
I15	I22	I70.6	E13.4		E78.4	Z86.32
	I23	I70.7	G60		E78.5	O24
	I24.1	I70.9				
	I24.8	E08.51				
	I24.9	E08.52				
	I25	E08.59				
	Z95.1	E09.51				
	Z95.5	E09.52				
		E09.59				
		E10.51				
		E10.52				
		E10.59				
		E11.51				
		E11.52				
		E11.59				
		E13.51				
		E13.52				
		E13.59				
