# Supplementary material for: Predicting Current Glycated Hemoglobin Values in Adults: Development of an Algorithm From the Electronic Health Record
Source: JMIR Med Inform. 2018 Oct 22;6(4):e10780. doi: 10.2196/10780 (PMC6231807; doi:10.2196/10780)
Supplement: Multimedia Appendix 2 [file medinform_v6i4e10780_app2.pdf]

Multimedia Appendix 2.

Table 1. Regression equation for predicting the probability of current glycated hemoglobin ≥5.7%.

**Intercept**				
	-	6.3471		
**Random Blood Glucose (glu.random) in mg/dL**				
	+	0.0071	*	glu.random
	+	2.7646e-05	*	max(glu.random - 74, 0)**3
	-	4.9472e-05	*	max(glu.random - 89, 0)**3
	+	2.1826e-05	*	max(glu.random - 108, 0)**3
**Smoking Status (smoking.status)**				
	+	0	*	(smoking.status = “Current Smoker”)
	-	0.3691	*	(smoking.status = "Former Smoker")
	-	0.4212	*	(smoking.status = "Never Smoker")
**Serum Non – High Density Lipoprotein (non.hdl) in mg/dL**				
	+	0.0163	*	non.hdl
	+	4.6091e-07	*	max(non.hdl - 92, 0)**3
	-	8.3318e-07	*	max(non.hdl - 134, 0)**3
	+	3.7227e-07	*	max(non.hdl - 186, 0)**3
**Body Mass Index (BMI) in Kg/m^2**				
	+	0.0627	*	BMI
	-	4.4558e-05	*	max(BMI - 22.305816, 0)**3
	+	7.2103e-05	*	max(BMI - 29.475615, 0)**3
	-	2.7544391e-05	*	max(BMI - 41.074144, 0)**3
**Estimated Glomerular Filtration Rate (eGFR) in mL/min/1.73m^2 [a]**				
	+	0.01025	*	eGFR
	-	1.5910e-06	*	max(eGFR - 50.516046, 0)**3
	+	3.7623e-06	*	max(eGFR - 95.739809, 0)**3
	-	2.1713e-06	*	max(eGFR - 128.87604, 0)**3
**Race**				
	+	0	*	(race = “Black or African American”)
	-	0.1033	*	(race = "Other")
	-	1.0373	*	(race = "White or Caucasian")
**Age in years**				
	+	0.0595	*	age
	-	1.2911e-05	*	max(age - 29.276934, 0)**3
	+	2.5892e-05	*	max(age - 49.620808, 0)**3
	-	1.2981e-05	*	max(age - 69.856263, 0)**3
**Serum Total Cholesterol (TC) in mg/dL**				
	-	0.0126	*	TC
	-	3.5965e-07	*	max(TC - 139, 0)**3
	+	6.5936e-07	*	max(TC - 184, 0)**3
	-	2.9971e-07	*	max(TC - 238, 0)**3

[a] eGFR was estimated using the CKD-Epi equation[38]
[b] Obesity diagnostic codes included: ICD9(278)and ICD10(E66)
max =maxima
e=exponential notation. For example: 2.4e-07 = 2.4 * 10^{-7}
= exponentiation. For example: 53 = 5^3

Table 2. Example application of the prediction model for two patient scenarios.

	**Patient 1**	**Patient 2**

**Age in years**	65	45
**Race**	Black or African American	White or Caucasian
**Body Mass Index**	26.8	21.6
**Smoking Status**	Former Smoker	Never Smoker
**eGFR**	31	89
**Random Blood Glucose**	119	87
**Non-HDL Cholesterol**	105	87
**Total Cholesterol**	143	162
**Linear Predictor (LP)**	0.64	-2.96
**Calculated Probability of HbA1c >5.7%**	0.65	0.05
**Actual HbA1C Value**	8.8	4.9

How to calculate probability

Probability of elevated HbA1c (>=5.7%) = exp(LP)/(1+exp(LP))

LP is the linear predictor, which can be obtained by calculating the formula in Table 3. For categorical variables: the appropriate cell is replaced with the number 1 while the other cells receive a value of 0.
